# Costs of diagnostic and preoperative workup with and without breast MRI in older women with a breast cancer diagnosis

**DOI:** 10.1186/s12913-016-1317-6

**Published:** 2016-02-27

**Authors:** Tracy Onega, Anna N.A. Tosteson, Julie Weiss, Jennifer Alford-Teaster, Rebecca A. Hubbard, Louise M. Henderson, Karla Kerlikowske, Martha E. Goodrich, Cristina O’Donoghue, Karen J. Wernli, Wendy B. DeMartini, Beth A Virnig

**Affiliations:** Department of Biomedical Data Science, Geisel School of Medicine at Dartmouth, Medical Center Drive, Lebanon, NH USA; Norris Cotton Cancer Center, Geisel School of Medicine at Dartmouth, Lebanon, NH USA; The Dartmouth Institute for Health Policy and Clinical Practice, Geisel School of Medicine at Dartmouth, Lebanon, NH USA; Department of Biostatistics and Epidemiology, University of Pennsylvania, Philadelphia, PA USA; Department of Radiology, University of North Carolina, Chapel Hill, NC USA; Departments of Medicine and Epidemiology and Biostatistics, University of California, San Francisco, CA USA; Department of Veterans Affairs, General Internal Medicine Section, University of California, San Francisco, CA USA; Department of Surgery, Moffit Cancer Center, Tampa, FL USA; Group Health Research Institute, Seattle, WA USA; Department of Radiology, University of Wisconsin School of Medicine and Public Health, Madison, WI USA; School of Public Health, University of Minnesota, Minneapolis, MN USA

**Keywords:** Beast cancer, Breast MRI, Diagnostic/preoperative breast MRI, and cost of breast MRI

## Abstract

**Background:**

Breast cancer in the U.S. - estimated at 232,670 incident cases in 2014 - has the highest aggregate economic burden of care relative to other female cancers. Yet, the amount of cost attributed to diagnostic/preoperative work up has not been characterized. We examined the costs of imaging and biopsy among women enrolled in Medicare who did and did not receive diagnostic/preoperative Magnetic Resonance Imaging (MRI).

**Methods:**

Using Surveillance, Epidemiology and End Results (SEER)- Medicare data, we compared the per capita costs (PCC) based on amount paid, between diagnosis date and primary surgical treatment for a breast cancer diagnosis (2005–2009) with and without diagnostic/preoperative MRI. We compared the groups with and without MRI using multivariable models, adjusting for woman and tumor characteristics.

**Results:**

Of the 53,653 women in the cohort, within the diagnostic/preoperative window, 20 % (*N* = 10,776) received diagnostic/preoperative MRI. Total unadjusted median costs were almost double for women with MRI vs. without ($2,251 vs. $1,152). Adjusted costs were higher among women receiving MRI, with significant differences in total costs ($1,065), imaging costs ($928), and biopsies costs ($138).

**Conclusion:**

Costs of diagnostic/preoperative workups among women with MRI are higher than those without. Using these cost estimates in comparative effectiveness models should be considered when assessing the benefits and harms of diagnostic/preoperative MRI.

**Electronic supplementary material:**

The online version of this article (doi:10.1186/s12913-016-1317-6) contains supplementary material, which is available to authorized users.

## Background

The high incidence of female breast cancer in the United States (U.S.) – estimated to affect 232,670 women in 2014 - [1] results in an aggregate economic burden of care relative to other cancers that is among the highest of all cancers in women [2]. While the majority of breast cancer treatment related costs occur in the first 12 months following diagnosis [2], costs for women as they are diagnosed and deciding upon initial diagnostic/preoperative treatment have not been characterized. Women with breast cancer and their providers face a number of decisions during the diagnostic and preoperative period, including which diagnostic testing, workup and primary treatment options they will pursue. Cost is one of several key factors cited by women with breast cancer as important in weighing their breast cancer care options [3].

**Fig. 1:**
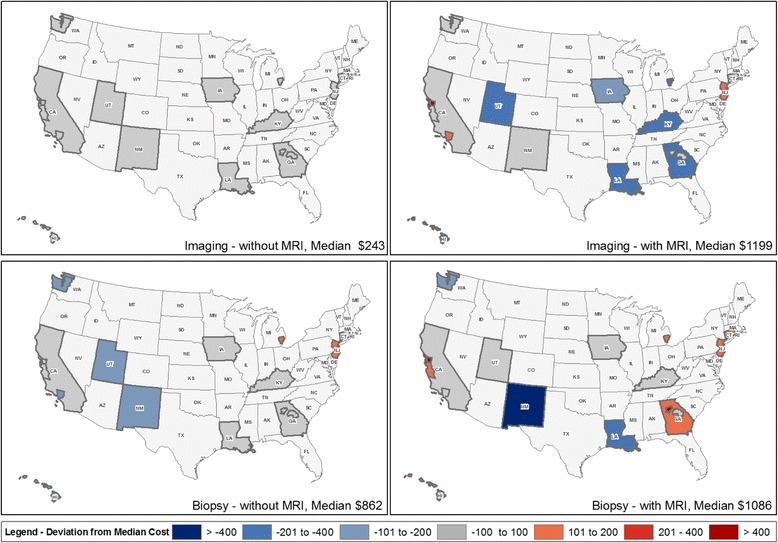
Diagnostic/preoperative imaging and biopsy costs, with and without MRI, among female Medicare beneficiaries with breast cancer in SEER registries (2005-2009)

Breast MRI has been reimbursed by Medicare and other payers since 1991for women with a breast cancer diagnosis, but only in the past 10 years has its use Fibecome widespread in clinical workup, increasing dramatically from an estimated 1 % in 2001 to between 21 and 50 % of breast cancer cases by 2010, with considerable regional variability [4–8]. The utility of breast MRI is its high sensitivity – especially compared to mammography – conferring an increased ability to determine extent of disease and detect occult cancer among women with a breast cancer diagnosis [9]. However, breast MRI also increases detection of benign and low malignant potential lesions, which often require further workup in order to make a final assessment [9]. This workup potentially leads to increased costs that may not be offset by other benefits. Preoperative MRI remains controversial in part due to: lack of evidence for improved outcomes, increased potential for false-positives, and the possibility of surgical overtreatment [10–13]. Given the increasing prevalence of breast MRI during the diagnostic/preoperative period even amidst uncertainty regarding harm and benefit tradeoffs, comparing effectiveness against mammography (+/− ultrasound) is an identified national priority [14].

Comparative effectiveness requires quantifying tradeoffs, a key dimension of which is cost, to determine the relative benefits and harms of alternative clinical practices for use by individuals and public policy makers [15]. In an era of increasingly constrained health care budgets, developing a clear understanding of costs weighed against clinical benefits is critical to rational resource use [15]. Fortunately, evidence of the clinical impacts of preoperative breast MRI is accumulating rapidly. Specifically, results from randomized controlled trials and observational studies are providing insights into preoperative MRI effects on cancer detection, biopsy rates, biopsy yield, surgical treatment, and recurrence [4–6, 8, 16–21]. To date, there have been no reports of the impact of diagnostic/preoperative breast MRI on aggregate economic considerations particularly compared to standard workup involving mammography with or without ultrasound. Although the cost of a breast MRI is about 10-times higher than a diagnostic mammogram [22], the overall costs of workups including breast MRI are not known and are relevant both for women and for payers.

The objective of this study was to examine per capita costs (PCC) of imaging and biopsy in the diagnostic/preoperative window among women enrolled in Medicare who did and did not receive MRI during their breast cancer diagnosis and preoperative workups. To address this objective, we used a woman-centered framework rather than societal, by measuring costs as the amount actually paid for services, rather than a nationally-standardized cost estimate. Thus, our aim was to estimate costs at the woman level to provide estimates when considering tradeoffs in the use of breast MRI. The overall goal is to inform the current practice and policy debates over use of diagnostic/preoperative MRI from an economic standpoint to help guide clinically appropriate use of this rapidly diffusing technology [4].

## Methods

### Data source

Surveillance, Epidemiology and End Results (SEER)-Medicare data were used for this study (2004–2010). The SEER program includes 17 population-based cancer registries representing about 24 % of the U.S. population that linked to Medicare administrative and health care claims data [22]. The SEER-Medicare data have been used to study health disparities, quality of care and cost of care across the cancer control continuum [23]. All patient data from the SEER-Medicare database used in this study were de-identified. The analysis plan, manuscript and tables were reviewed and approved by SEER- Medicare.

### Ethics, consent and permissions

The study was approved by the Committee for Protection of Human Subjects at Dartmouth College.

### Study population

The study cohort included women aged 66 yrs. or older at the time of an incident breast cancer diagnosis in 2005–2009 who were enrolled in Medicare with equal parts A and B enrollment and not enrolled in an HMO for one year before and six months after breast cancer diagnosis (*N* = 71,193). Women were excluded if: they lacked a pathologic breast cancer diagnosis confirmation, the diagnosis source was a nursing home, they did not receive cancer directed surgery, or no diagnostic biopsy could be found (*N* = 7,248). Lastly, we required the woman’s primary surgical treatment with either mastectomy or breast conserving surgery (BCS) to occur within six months of the diagnosis date (*N* = 7,496), resulting in a final cohort of 56,449 women.

### Definitions

The Medicare claim files (Outpatient and Carrier) were used to obtain the biopsy at time of diagnosis, the primary surgical treatment, and comorbidities using Current Procedural Terminology (CPT)/Healthcare Common Procedure Coding System (HCPCS) and International Classification of Diseases (ICD-9) diagnosis and procedure codes (Additional file 1: Appendix I). Comorbidities were defined by applying the Klabunde adaptation of the Charlson Index [24]. Since the SEER cancer diagnosis date is defined only up to month and year, we determined the breast cancer *diagnosis date* (month, day and year) from Medicare claims defined as the biopsy claim date closest to the SEER diagnosis date. The time between the initial breast imaging or biopsy within 60 days prior to diagnosis and the primary surgical treatment was defined as the *diagnostic/preoperative window*. Within the diagnostic/preoperative window, we also distinguished two periods: 1) the diagnostic period, defined as 60 days prior to, and including, the diagnosis date; 2) the preoperative period, defined as: post diagnosis date to initial surgery date. The Outpatient and Carrier claim files were used to capture *breast imaging* (MRI, Ultrasound, and Mammography) and *biopsies* during the diagnostic/preoperative window. We identified women as receiving diagnostic/preoperative MRI if receipt of MRI occurred anytime during the diagnostic/preoperative window. We included MRI(s) prior to diagnosis since breast MRI performed during the diagnostic period could potentially be used in preoperative evaluation.

### Costs (Outcome)

Costs associated with breast cancer care services delivered during the diagnostic/preoperative window were obtained from the Medicare Carrier and Outpatient files and were defined by *payment* amounts. We included all costs associated with breast cancer care service claims (e.g. all line items per claim for breast imaging and/or biopsy). To calculate costs for each woman, we took the sum of the line allowed amount variable from the Carrier file (lalowamt) and the revenue center payment amount and revenue center patient responsibility payment amount from the Outpatient file (pay and ptntresp) over the diagnostic/preoperative window. These variables combine to represent the total paid for services of interest. We focus on total payments and do not differentiate between payments from Medicare and payments from other sources such as the patient, Medigap policies or Medicaid.

### Patient and cancer tumor characteristics

For patient and tumor characteristics of interest, we used the SEER PEDSF (Patient Entitlement and Diagnosis Summary File) to identify age at diagnosis, race, urban/rural residence, SEER registry, year of diagnosis, and quartiles of median household income for census tract, stage, histology, grade, nodal status, estrogen receptor (ER) status, and size. Age at diagnosis was categorized as follows (in years): 66–69, 70–74, 75–79, 80–84, and 85+. Race was categorized based on self-report within the Medicare enrollment database and was categorized as “White”, “Black”, or “Other”, using the categorical value provided within the PEDSF. The category “Other” was collapsed due to small numbers within the other race categories of: Asian, Hispanic, North American Native, and Unknown. Urban versus rural residence was provided in the PEDSF based on patient residence at the time of diagnosis. Quartiles of median household income for census tract of residence at the time of diagnosis were derived empirically from the study population. All of the cancer/tumor characteristics were taken from the PEDSF, based on SEER registry data, and included: stage at diagnosis (0-IV), histology (ductal, lobular, mixed, and other), grade (high, intermediate, low), nodal status, (positive, negative), ER status (positive, negative), an size in cm (<1, 1 to <2, 2 to <5, and 5+). We dichotomized time from diagnoses to primary surgical treatment at the sample median.

### Statistical analysis

We report median and interquartile range (IQR) of PCC among women who received breast cancer care services during the diagnostic/preoperative window by patient and tumor characteristics. We mapped the median amount ($) costs exceeded or fell below the overall median value for each SEER registry. Imaging alone and biopsy alone services were mapped separately. Mapping was performed using ArcGIS v10.1 with source geography downloaded from the NCI GIS portal [25].

We used a multivariable regression model to compare total PCC between the diagnostic/preoperative MRI groups, adjusting for age, race, residential location, census tract median income, SEER registry, comorbidities, year of diagnosis, stage at diagnosis, grade, nodal status, estrogen receptor status, tumor size, and time from diagnosis to primary surgical treatment. We estimated differences in adjusted mean costs using generalized linear models (GLMs) with gamma distributed errors, identity link function, and robust variance estimators to account for the anticipated violation of the assumption of the gamma mean-variance relationship common in cost data. The model was repeated separately for the diagnostic period and preoperative period described above. Adjusted costs for each MRI group in the diagnostic and preoperative periods were estimated from the fitted GLMs by using predictive margins to standardize estimated mean costs in each group to the overall distribution of patient and tumor characteristics observed in the study sample included in each model [26]. Analyses were conducted in SAS (SAS 9.3 System Options: Reference, Second Edition. Cary, NC: SAS Institute Inc.; 2011) and Stata/SE 12.1 (StataCorp. 2011. Stata 12 Base Reference Manual. College Station, TX: Stata Press).

## Results

Among the 56,449 women in the study cohort, a breast imaging claim (mammogram, ultrasound or an MRI) for 53,653 women was found within the diagnostic/preoperative window. Of the 53,653, 20 % (*N* = 10,776) received an MRI. Among the women defined in the MRI group (*N* = 10,776), 1,713 women had an MRI in the diagnostic period (16 %) and 9,196 women had an MRI in the preoperative period (84 %). 133 women had an MRI in both periods (12 %).

The total unadjusted median diagnostic/preoperative costs were almost double for women with a diagnostic/preoperative MRI vs. without ($2,251 v. $1,152; Table 1). Higher total median costs were found for each increase in median income quartile, for urban versus rural, for each successive year of diagnosis, for lower staged tumors, smaller tumors, tumors with a positive nodal status, and for women whose initial therapy was breast conserving surgery (BCS) with radiation compared to those with mastectomy or BCS alone (Tables 1 and 2). Older age, increasing comorbidities, and having primary surgical treatment closer to diagnosis were associated with lower median costs (Tables 1 and 2). Similar trends were found among the diagnostic/preoperative breast MRI groups.

**Table 1:** Among the female SEER-Medicare breast cancer cohort^a^ (2005–2009; *N* = 56,449), median and interquartile range (IQR) per capita costs for women within the diagnostic/preoperative window (*N* = 53,653) by patient characteristics, with and without breast MRI

	Total Median Costs				MRI		No MRI	
	(*N* = 53,653)				(*N* = 10,776)		(*N* = 42,877)	
Characteristics^b^ of women	N	%	Median	IQR ($)	Median	IQR ($)	Median	IQR ($)
Total	53,653	100 %	$1,353	(818–2,042)	$2,251	(1,783–2,971)	$1,152	(708–1,727)
Age at diagnosis (yrs.)								
66–69	11,130	21 %	$1,553	(969–2,268)	$2,307	(1,810–3,097)	$1,241	(778–1,821)
70–74	13,949	26 %	$1,447	(892–2,146)	$2,271	(1,806–3,045)	$1,211	(760–1,781)
75–79	12,713	24 %	$1,362	(837–2,036)	$2,223	(1,759–2,934)	$1,178	(733–1,767)
80–84	9,633	18 %	$1,220	(734–1,871)	$2,201	(1,733–2,742)	$1,082	(668–1,654)
85+	6,228	12 %	$1,024	(608–1,625)	$2,057	(1,660–2,687)	$948	(576–1,459)
Race								
White	47,150	89 %	$1,360	(827–2,046)	$2,243	(1,785–2,955)	$1,154	(711–1,722)
Black	3,732	7 %	$1,293	(757–1,946)	$2,272	(1,674–3,288)	$1,162	(697–1,779)
Other^c^	2,741	5 %	$1,317	(761–2,065)	$2,331	(1,799–3,025)	$1,099	(677–1,711)
Residential location								
Urban	48,332	90 %	$1,368	(829–2,071)	$2,264	(1,796–2,986)	$1,154	(717–1,732)
Rural	5,311	10 %	$1,224	(672–1,790)	$2,040	(1,603–2,708)	$1,134	(613–1,686)
Census Tract Median Income Quartiles								
1st (<=$36,000)	13,336	25 %	$1,216	(697–1,818)	$2,045	(1,622–2,652)	$1,106	(637–1,674)
2nd ($36,001–$47,500)	13,345	25 %	$1,287	(781–1,985)	$2,216	(1,751–2,942)	$1,123	(695–1,709)
3rd ($47,501–$64,700)	13,409	25 %	$1,385	(852–2,094)	$2,239	(1,790–2,958)	$1,168	(742–1,748)
4th (> = $64,701)	13,065	25 %	$1,538	(959–2,287)	$2,387	(1,882–3,159)	$1,227	(778–1,812)
Comorbidities								
0	33,742	63 %	$1,390	(847–2,088)	$2,256	(1,786–2,974)	$1,160	(726–1,730)
1	12,889	24 %	$1,324	(797–2,003)	$2,240	(1,760–2,965)	$1,157	(703–1,741)
2+	7,022	13 %	$1,227	(712–1,894)	$2,231	(1,759–2,956)	$1,099	(654–1,686)
Year of Diagnosis								
2005	10,516	20 %	$1,155	(648–1,765)	$2092	(1,368–2,864)	$1,070	(614–1,641)
2006	10,863	20 %	$1,211	(706–1,871)	$2134	(1,674–2,851)	$1,074	(649–1,653)
2007	10,645	20 %	$1,268	(766–1,949)	$2131	(1,687–2,805)	$1,073	(674–1,585)
2008	10,777	20 %	$1,533	(966–2,188)	$2285	(1,842–2,975)	$1,252	(836–1,761)
2009	10,852	20 %	$1,674	(1,065–2,368)	$2389	(1,904–3,171)	$1,316	(924–1,966)

^a^Enrollment based on age, fee-for-service, and equal parts A and B

^b^Missing (N): Race (30), Residential location (20), Median income (498)

^c^Other includes Hispanic, Asian, Native American and Other

**Table 2:** Among the female SEER-Medicare breast cancer cohort^a^ (2005–2009; *N* = 56,449), median and interquartile range (IQR) per capita costs for women within the diagnostic/preoperative window (*N* = 53,653) by tumor characteristics, with and without breast MRI

	Total Median Costs				MRI		No MRI	
	(*N* = 53,653)				(*N* = 10,776)		(*N* = 42,877)	
Characteristics^b^ of women	N	%	Median	IQR ($)	Median	IQR ($)	Median	IQR ($)
Total	53,653	100 %	$1,353	(818–2,042)	$2,251	(1,783–2,971)	$1,152	(708–1,727)
Stage								
0	8,533	16 %	$1,721	(1,232–2,368)	$2,627	(2,090–3,228)	$1,524	(1,160–2,085)
I	25,146	47 %	$1,393	(878–2,080)	$2,255	(1,799–2,985)	$1,190	(781–1,751)
II	14,328	27 %	$1,106	(627–1,838)	$2,093	(1,662–2,776)	$891	(547–1,442)
III	3,838	7 %	$1,008	(535–1,76)	$2,078	(1,631–2,775)	$825	(452–1,345)
IV	503	1 %	$927	(449–1,685)	$2,023	(1,690–2,791)	$738	(405–1,294)
Unknown	1,305	2 %	$1,310	(815–1,938)	$2,118	(1,620–2,743)	$1,176	(741–1,712)
Histology (invasive)								
Ductal	33,134	62 %	$1,259	(751–1,953)	$2,186	(1,742–2,892)	$1,068	(664–1,641)
Lobular	5,005	9 %	$1,359	(765–2,061)	$2,155	(1,736–2,837)	$1,025	(615–1,576)
Mixed	3,177	6 %	$1,346	(795–2,094)	$2,264	(1,774–2,968)	$1,058	(656–1,580)
Other	3,804	7 %	$1,232	(714–1,877)	$2,178	(1,623–2,927)	$1,078	(648–1,674)
Grade								
High	12,125	24 %	$1,392	(877–2,083)	$2,284	(1,811–3,050)	$1,191	(772–1,754)
Intermediate	22,522	45 %	$1,351	(822–2,054)	$2,238	(1,776–2,944)	$1,129	(708–1,715)
Low	15,133	30 %	$1,289	(726–1,969)	$2,212	(1,747–2,899)	$1,100	(636–1,676)
Nodal Status								
Positive	11,009	25 %	$1,119	(623–1,851)	$2,113	(1,652–2,842)	$890	(534–1,439)
Negative	32,952	75 %	$1,379	(847–2,074)	$2,242	(1,788–2,951)	$1,158	(739–1,734)
ER Status								
Positive	40,999	84 %	$1,367	(827–2,061)	$2,249	(1,787–2,971)	$1,152	(716–1,732)
Negative	7,978	16 %	$1,263	(728–1,960)	$2,231	(1,747–2,935)	$634	(634–1,633)
Size								
<1 cm	12,455	25 %	$1,569	(1,090–2,253)	$2,451	(1,967–3,184)	$1,369	(987–1,936)
1 to <2 cm	19,135	39 %	$1,348	(824–2,031)	$2,187	(1,745–2,925)	$1,127	(723–1,716)
2 to <5 cm	15,281	31 %	$1,098	(610–1,826)	$2,097	(1,660–2,798)	$891	(532–1,459)
5+ cm	2,620	5 %	$1,057	(535–1,870)	$2,133	(1,719–2,810)	$840	(438–1,420)
Primary Treatment								
Mastectomy	19,650	37 %	$1,267	(696–1,955)	$2,265	(1,746–3,028)	$1,053	(602–1,686)
BCS^c^ without Radiation	12,972	24 %	$1,362	(838–2,035)	$2,285	(1,782–2,955)	$1,198	(744–1,744)
BCS^c^ with Radiation	21,031	39 %	$1,413	(909–2,126)	$2,230	(1,801–2,925)	$1,201	(602–1,762)
Number of days in Diagnostic/preoperative Window								
<= Median (25 days)	27,779	52 %	$1,172	(685–1,768)	$2,009	(1,626–2,543)	$1,056	(633–1,588)
> Median (25 days)	25,874	48 %	$1,593	(998–2,354)	$2,392	(1,878–3,229)	$1,272	(828–1,915)

^a^Enrollment based on age, fee-for-service, and equal parts A and B

^b^Missing (N): Grade (3,873), Nodal status (9,692), ER status (4,598), Size (4,162)

^c^BCS: Breast Conserving Surgery

Among workups not including MRI, negligible deviations from the median imaging costs were observed by SEER registry. However, in the MRI group, wider positive imaging cost deviations were found in the SEER registries of San Francisco, Los Angeles, and New Jersey. Variation in the total median costs for SEER registries are presented geographically as four distinct maps divided by receipt of biopsy or breast imaging and separately by receipt of MRI (Fig. 1). The median biopsy costs were somewhat higher among the MRI group ($1,086 v. $862; MRI vs. no MRI), but there were markedly lower biopsy costs in the New Mexico and the Seattle SEER registries for both diagnostic/preoperative groups (MRI v. no MRI). Compared with other regions in the diagnostic/preoperative MRI group, the highest biopsy median costs were found in Atlanta and San Francisco. For those without diagnostic/preoperative MRI, the highest biopsy costs were found in Detroit and New Jersey.

The unadjusted and adjusted (patient and tumor characteristics) PCCs were similar overall and among the diagnostic/preoperative MRI groups (Table 3). The total adjusted cost difference between the MRI groups was $1,065. Partitioning total costs by imaging and biopsy costs, we found the difference was mainly attributable to costs of imaging ($928).

**Tables 3:** Total crude (*N* = 53,653) and adjusted breast cancer per capita costs, with and without breast MRI and stratified by the diagnostic and preoperative periods, among SEER-Medicare females (2005–2009)

		Total Costs			MRI		No MRI		MRI vs. No MRI
	N^a^	N^b^	PCC^c^	Adjusted PCC^b,c^	PCC^c^	Adjusted PCC^b,c^	PCC^c^	Adjusted PCC^b,c^	Difference^d^
Total	53,653	43,414	$1,560	$1,541	$2,524	$2,382	$1,318	$1,317	$1,065
Imaging costs	53,653	43,414	$455	$467	$1,227	$1,200	$261	$272	$928
Biopsy costs	53,653	43,414	$1,105	$1,074	$1,297	$1,183	$1,057	$1,045	$138
Diagnostic period	53,653	43,414	$1,127	$1,107	$1,313	$1,253	$1,080	$1,069	$185
I maging costs	52,487	42,491	$288	$293	$442	$418	$250	$259	$159
Biopsy costs	53,653	43,414	$845	$821	$884	$845	$835	$814	$31
Preoperative period	20,570	16,775	$1,131	$1,127	$1,356	$1,316	$932	$947	$369
Imaging costs	14,834	12,358	$627	$508	$912	$907	$127	$128	$779
Biopsy costs	11,947	9,504	$1,169	$1,165	$1,282	$1,173	$1,122	$1,158	$15

^a^Unadjusted N

^b^Model Ns after excluding patients with missing patient or tumor characteristics

^c^PCC: Per Capita Costs

^d^Difference between the MRI groups from the adjusted models

Of the 38 % women (*N* = 20,570) who had at least one breast event (MRI, Mammogram, Ultrasound or Biopsy) during the preoperative period, 72 % (14,834/20,570) of the women had an imaging event and 58 % (11,947/20,570) had a biopsy. Within the diagnostic/perioperative window, we found that in the diagnostic period, the cost difference between the MRI and no MRI groups was $185 ($159 for imaging and $31 for biopsy); in the preoperative period, the difference was $369 ($779 for imaging and $15 for biopsy; Table 3).

## Discussion

Our study is the first to report diagnostic/preoperative costs for breast cancer in the Medicare population, and to compare those costs between women with and without diagnostic/preoperative MRI in their clinical workup. We found that the total adjusted PCC for the diagnostic/preoperative period were $1,541 and were nearly double for women with diagnostic/preoperative MRI ($2,382) compared to without ($1,317).

Diagnostic/preoperative costs did not differ dramatically over the study period, but decreased with more comorbidities, higher stage, and larger tumors. Geographic variation in costs was greatest for biopsies, regardless of receipt of diagnostic/preoperative MRI, and was very low for imaging without diagnostic/preoperative MRI. Overall, we found that diagnostic/preoperative MRI was associated with higher PCCs by an estimated $1,065 after adjusting for patient and tumor characteristics. However, the difference in imaging costs ($928) between those with and without diagnostic/preoperative MRI was considerably higher than the difference in biopsy costs ($138). Cost differences between the MRI v. no MRI groups were more notable in the preoperative period compared to the diagnostic period, which seems largely attributable to the cost of MRI itself. The national payment amount for a bilateral MRI found in the physician fee schedule (ref: http://www.cms.gov/apps/physician-fee-schedule/search/) ranged from $994 to $905 in the years 2007 to 2009; hence the imaging costs difference of $928 found across the years in this study appears to be due to the cost of an MRI.

Biopsy cost differences were minimal in both the diagnostic and preoperative periods. Thus, use of MRI in diagnostic and preoperative workup for breast cancer does not appear to lead to excess costs beyond the cost of the MRI exam. These differences between the diagnostic/preoperative MRI groups as well as the cost trends observed in the geographic, patient and tumor characteristics provide an economic perspective of the costs attributed to diagnostic/preoperative workup.

Our study is difficult to compare to prior literature, given the lack of cost estimates for breast imaging and biopsies in prior studies. However, estimating diagnostic/preoperative costs in breast cancer overall, and in relation to use of diagnostic/preoperative MRI is important for several reasons as cost measures: 1) provide critical inputs for comparative effectiveness research to guide clinical decision making; 2) facilitate consumer-centered public cost reporting; and 3) inform decisions about costs once a breast cancer diagnosis occurs which may influence women’s choices of initial treatment [3]. This study fills an important gap in our current understanding of the cost implications of diagnostic/preoperative breast MRI. That is, while not conclusive, most studies to date have not reported improved outcomes from diagnostic/preoperative breast MRI, particularly for reoperation and recurrence [5, 27–29]. Without clinical benefit, the increased costs associated with diagnostic/preoperative MRI might be interpreted as harmful to women and health care systems. The results presented here provide cost estimates, which can be combined with measures needed to model harms from women’s perspectives and which will be evaluated in relation to any benefits, in comparative effectiveness models.

Public reporting of costs is an important component of women’s ability to be knowledgeable about the likely cost of breast cancer care and to make informed choices. The Agency for Healthcare Research and Quality (AHRQ) reported a general lack of consumer-centered cost reporting, which is a necessary component for women to choose high-value health care (when linked to quality) [14]. As Keselman et al. note, patients need data to inform their decisions to be effective health care consumers [30].

Relatedly, even though diagnostic/preoperative care is only a portion of the costs of cancer care [2, 31, 32], concern over costs once a diagnosis occurs may influence women’s choices of initial treatment [3]. Qualitative evidence has revealed financial concerns and monetary stress for women newly diagnosed with breast cancer [3], which suggests that cost may play a role in planning initial treatment and breast cancer management.

Although this cross-sectional study was large, population-based, and used publicly available data, several limitations should be noted. First, we did not account for type of biopsy, such as core versus excisional, which is likely to be associated with variation in cost. Separate studies are currently underway to fully characterize detailed aspects of diagnostic/preoperative biopsy use in women with and without diagnostic/preoperative MRI. Furthermore, because claims data may be inexact with respect to date of service and date of claim, we performed a sensitivity analysis of our definition of diagnostic/preoperative window to determine whether biopsies performed to establish the diagnosis may be classified as diagnostic/preoperative. Our analyses were robust to the assessment of the ‘start’ of the diagnostic/preoperative window based on the putative diagnostic biopsy. Also, as in all Medicare claims-based studies, the potential for unmeasured confounding exists, and our findings are only generalizable to the Medicare population. Patterns of use and costs may be different for women younger than the >65 yrs. old included in this study. Another limitation of this claims-based analysis is the lack of information on women’s preferences and clinical decision making related to breast cancer care. Finally, although not a limitation, we do note that our cost measure approach (per capita based on amount paid) is one of several methods to evaluate cost. Another common approach is to use a standardized national estimate for each type of service and apply those costs to the services identified in claims. The per capita amounts paid costs measures used in this study are likely to be more useful at the individual woman level for informing clinical decisions. The standardized national estimate approach may be better used if one seeks to compare health care systems, do small area analysis, or inform the societal perspective. Both of these two methods are acceptable, but are different “lenses” through which to interpret costs.

## Conclusion

Since we found differences in total costs between the MRI groups, the results suggest the excess costs associated with diagnostic/preoperative MRI could be factored into women’s decisions as a potential harm (with the marginal increase estimated at about $1,065). These estimates may prove useful for those engaged in comparative effectiveness studies of breast cancer diagnosis and for women and their healthcare providers as they consider alternative care pathways.

## Additional file

Additional file 1:
**Appendix 1 and 2.** (DOCX 300 kb)

## Abbreviations

MRI: Magnetic Resonance Imaging
PCC: Per Capita Costs
SEER: Surveillance, Epidemiology and End Results program
IRQ: Interquartile Range
GLMs: generalized linear models
AHRQ: Agency for Healthcare Research and Quality
