# Supplementary material for: Costs of diagnostic and preoperative workup with and without breast MRI in older women with a breast cancer diagnosis
Source: BMC Health Serv Res. 2016 Feb 27;16:76. doi: 10.1186/s12913-016-1317-6 (PMC4769533; doi:10.1186/s12913-016-1317-6)
Supplement: Additional file 1: — Appendix 1 and 2. (DOCX 300 kb) [file 12913_2016_1317_MOESM1_ESM.docx]

Appendix I. Procedure codes used in this analysis

| **Procedure** | **ICD^1^-9 Procedure Codes** | **CPT/HCPCS^1^ Codes** |
| --- | --- | --- |
| MRI |  | 76093, 76094, 76498, 77058, 77059, 0159T, C8903-C8908 |
| Mammogram | 87.37, 793.80, 793.82, V76.11, V76.12 | 76082, 76083, 76085, 76090-76092, 77051, 77052, 77055-77057, 3014F, 3340F-3345F, 3350F, 5060F, 5062F, 7020F,7025F, G0202-G0207, G0236, G8111-G8114, S8075 |
| Ultrasound | 88.73 | 76645 |
| Biopsy | 40.11, 40.22,40.3, 40.51, 85.1, 85.11, 85.12, 85.19, 85.2-85.25, 85.31, 85.32, 85.91, 85.99, 87.35 | 10022, 19000, 19001, 19030, 19100-19103, 19105, 19110, 19112, 19120-19126, 19260, 19271,19272, 19290-19295, 19499, 38500, 38505, 38510, 38525, 38530, 38740, 38792, 76087-76089, 76095-76098, 76360, 76393, 76942, 77012, 77021, 77031, 77032, 77053, 77054, 0046T, 0047T |
| Breast Conserving Surgery | 85.22, 85.23 | 19120, 19160, 19162, 19301, 19302 |
| Mastectomy | 85.33-85.36, 85.4, 85.41-85.48, V51.0 | 19180, 19182, 19200, 19220, 19240, 19303-19306 |

^1^Abbreviations: CPT – Current Procedural Terminology; ICD9-Internationsl Classification of Diseases, 9^th^ Edition; HCPCS - Healthcare Common Procedure Coding System


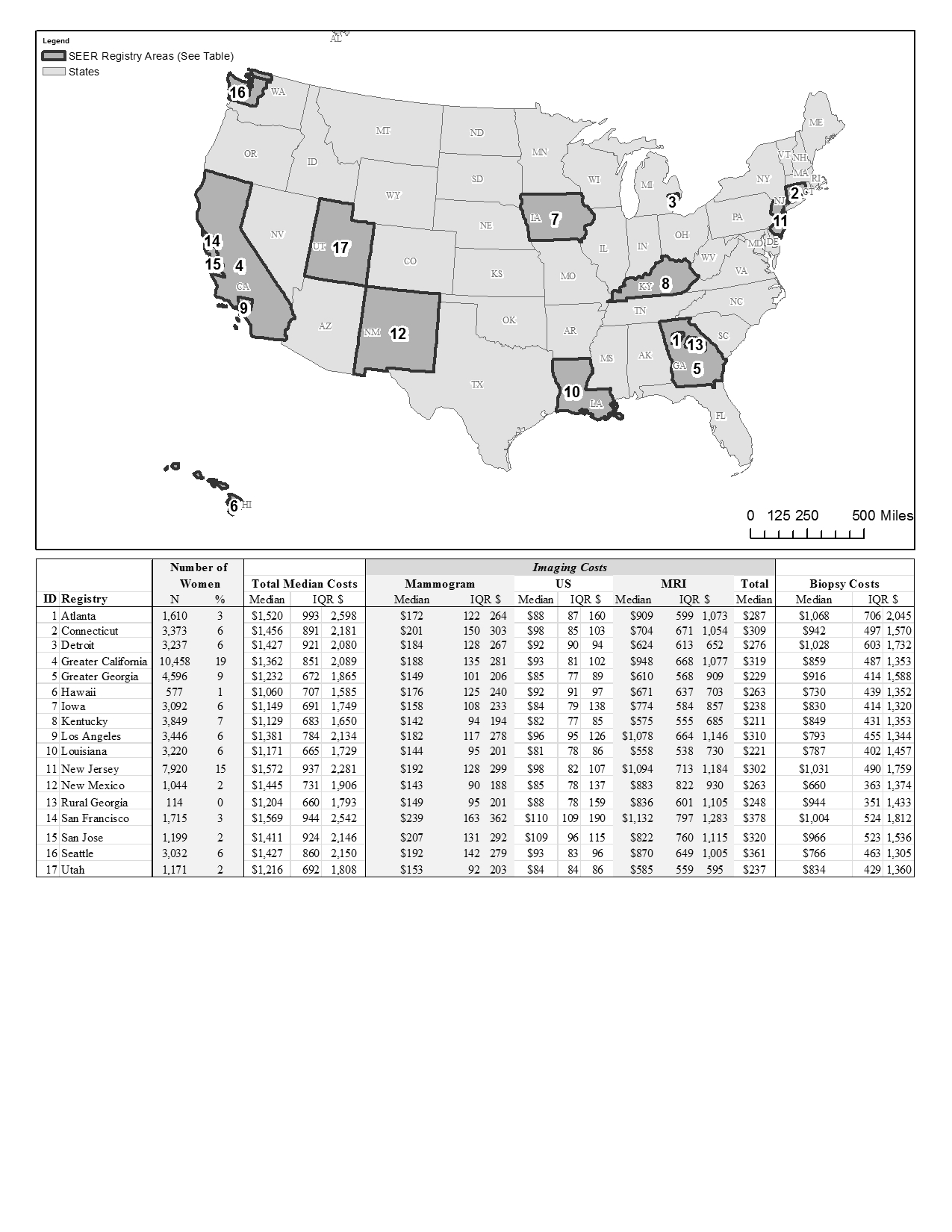
Appendix II. Summary of imaging and biopsy costs during the diagnostic/preoperative window for female Medicare beneficiaries in SEER registries (2005-2009)
